# Exploring the impact of mobile and migrant populations on mass drug administration coverage and effectiveness in Africa: A scoping review protocol

**DOI:** 10.1371/journal.pone.0324949

**Published:** 2025-05-29

**Authors:** Moussa Sangare, Yaya Ibrahim Coulibaly, Prithi Ravichandran, Abdoul Fatao Diabate, Claudia Duguay, Carol Vlassoff, Manisha A. Kulkarni, Alison Krentel

**Affiliations:** 1 International Center of Excellence in Research in Mali, University of Sciences, Techniques, and Technologies of Bamako, Bamako, Mali; 2 Interdisciplinary School of Health Sciences, University of Ottawa, Ottawa, Ontario, Canada; 3 Bruyère Health Research Institute, University of Ottawa, Ottawa, Ontario, Canada; 4 School of Epidemiology and Public Heath, University of Ottawa, Faculty of Medicine, Ottawa, Ontario, Canada; Yale University Department of Epidemiology and Public Health: Yale University School of Public Health, UNITED STATES OF AMERICA

## Abstract

**Background:**

Neglected tropical diseases (NTDs) affect populations in tropical regions, particularly low- and middle-income countries with limited economic and health resources. Mass drug administration (MDA) is a strategy for controlling and eliminating NTDs by treating entire at-risk populations to reduce parasite loads, interrupt transmission, and prevent reinfection. It is cost-effective, and promotes equity by reaching underserved communities. MDA is a critical approach to controlling and eliminating NTDs. Mobile populations in Africa such as nomadic groups and internally displaced persons, may lack access to MDA, which poses challenges to NTD elimination. This study aims to explore the influence of population mobility on the implementation, effectiveness, and sustainability of MDA in Africa.

**Materials and methods:**

This scoping review adheres to the PRISMA extension for scoping reviews and Joanna Briggs Institute (JBI) methodology. PCC (Population, Concept, Context) serves as the foundation for the study. Relevant papers published after 2000 will be identified through a comprehensive search of Medline Ovid, Embase, Web of Science, and gray literature. Studies addressing challenges to MDA in Africa’s and related to mobile populations will be included. An Excel spreadsheet modified from the JBI will be used for data extraction and analysis.

**Conclusion:**

The results of this review will shed light on how MDA coverage is affected by the phenomenon of mobile and migrant populations and what effective approaches, if any, have been used to address this problem and improve overall population access to MDA.

## Introduction

### Background and rationale

Neglected tropical diseases (NTDs) are a group of diseases and conditions that prevail in tropical and subtropical areas mainly in low- and middle-income countries, where inadequate sanitation and poor health services are common [1]. NTDs can cause illness, physical and mental suffering, disability, and stigma, decrease productivity and negatively affect children’s education [2]. Several clinical manifestations are associated with NTDs. For example, lymphatic filariasis (LF) may cause elephantiasis and hydrocele which are highly disfiguring and painful conditions for those affected [3]. Leprosy and leishmaniasis cause disfiguring, and stigmatizing scars and can lead to mental distress in affected patients [4] while urogenital schistosomiasis causes male and female genital lesions causing immeasurable suffering and shame, especially in Africa [5,6]. Other NTDs, such as onchocerciasis, can remain asymptomatic for years before complications emerge, resulting in a delayed diagnosis and more severe outcomes like river blindness [2].

The five most prevalent NTDs worldwide include: LF, onchocerciasis, schistosomiasis, soil-transmitted helminths (ascariasis, trichuriasis, and hookworm infection) and trachoma. These diseases can be controlled by preventive chemotherapy (PC-NTDs) delivered through mass drug administration (MDA) [7]. MDA is a safe and inexpensive means of delivery of essential drugs based on the principles of preventive chemotherapy, where populations or sub-populations are offered treatment regardless of their disease status [8]. These recommendations are based on the epidemiology of NTDs, host-parasite interaction, patient acceptability and drug effect [9,10].

Significant progress has been made in reducing the transmission and burden of NTDs worldwide through PC-NTDs [11,12]. In Africa, MDA and other control efforts have recently led to certification of national elimination of LF in Malawi [13] and Togo [14] bringing the total number of countries globally with LF elimination status to nineteen (19) [15]. In Mali, trachoma was eliminated in 2023 [16] and for onchocerciasis, Mali was successful at the pre-stop MDA survey in 2019 [17]. The pre-stop MDA survey assesses key epidemiological and entomological indicators to determine if treatment can be safely stopped without risking disease resurgence.

Despite these achievements, there remain many population groups or geographic areas that have not been reached with MDA interventions [18,19]. Not only does this contribute to inequity for those left untreated, but it also compromises the achievement of NTD elimination. Elimination goals remain in danger if at-risk groups like mobile and migrant populations (MMPs) (including nomadic pastoralist communities, internally displaced persons (IDPs)) routinely miss MDA, potentially contributing to hotspots that could lead to disease re-emergence [20]

The control and elimination of NTDs requires the treatment of all eligible individuals in all population groups, following the principle that no one should be left behind. Over the past decade, the risks and vulnerabilities faced by migrant and mobile populations in Africa have been compounded. For example, in the Sahel region, armed conflict, terrorism, poverty, violence, and growing insecurity have all contributed to an increased influx of people on the move [21]. MMPs are a significant proportion of the population in the African region [22,23]. Insecurity and exacerbated poverty are responsible for mobility that involves IDPs, nomadic pastoralists, refugees, migrants, among others. These individuals have been shown to have the least access to MDA when compared to the general population [24]. Climate change and severe weather events such as droughts, floods, and extreme heat are driving increased mobility as communities are forced to migrate/move in search of water, arable land, and livelihood opportunities. These movements often heighten vulnerabilities by disrupting access to essential health services particularly through interruptions in continuity of care. These movements also create health challenges in both the home and host communities that people leave and those they move to as mobile populations may miss essential preventative health measures, such as immunizations or antenatal care, due to constant movement in the home and host districts. In the host district, an influx of people can overwhelm local health facilities, resulting in longer wait times, shortages of medical supplies, and pressure on healthcare personnel [25].

Today, little is known about how best to provide essential health care to mobile and migrant populations. The constant mobility of populations marginalizes them or even excludes them from health services. To address this issue among mobile populations living in vulnerable circumstances, it is essential to understand the context in which interventions can be effective in responding to these specific situations. What are the barriers and facilitators to accessing MDA for PC-NTDs among MMPs (nomadic pastoralists, migrant laborers, IDPs, and refugees) in Africa; Why are these populations moving? Where are these populations coming from or going to? What challenges are associated with the capacity and willingness of stakeholders, including the beneficiary populations, to successfully implement MDA in order to effectively control/eliminate NTDs and other infectious diseases?

### Objectives

To address these questions, we will conduct a scoping review to explore the influence of population mobility on the implementation of mass drug administration (MDA) for preventive chemotherapy neglected tropical diseases (PC-NTDs) in Africa. This review aims to explore the factors driving population movement and assess their consequences on health outcomes, particularly in relation to the implementation and effectiveness of MDA programs in Africa. We seek to explore three primary questions:

1. **Reasons for movement and patterns:** to understand the key factors driving the movement patterns of MMPs in sub-Saharan Africa that impact the implementation of MDA for PC-NTDs, including seasonal work, conflict, insecurity, and nomadic lifestyles.
2. **Consequences on MDA programs:** To determine the health-related impacts of these movements on MDA programs, including how mobile populations might miss treatments, the challenge of re-administering drugs, potential increased transmission of diseases, and the strain on health systems in Africa.
3. **Successful approaches documented**: to compile evidence of successful approaches shown to effectively improve MDA coverage among mobile populations in sub-Saharan Africa, with a focus on impact and outcome data.

### Expected anticipated results

#### Mobility patterns and barriers to MDA access.

− MMPs, including nomadic pastoralists, migrant laborers, IDPs, and refugees, will have lower MDA coverage compared to settled populations due to their movement patterns.
− Seasonal migration related to work, climate variability, or conflict will contribute to missed treatment opportunities and inconsistent MDA access.

#### Health impacts of population mobility.

− High population mobility will lead to lower adherence to MDA schedules, increasing the risk of sustained transmission of PC-NTDs among these populations.
− Inadequate adaptation of MDA delivery models to mobile populations will result in suboptimal health outcomes compared to more stable populations.

#### Stakeholder engagement and implementation challenges.

− Limited coordination among health authorities, community leaders, and mobile populations will hamper effective MDA implementation.
− MDA strategies that fail to account for mobility patterns will show reduced effectiveness, leading to gaps in NTD control and elimination efforts.

#### Successful MDA approaches and coverage improvement.

− Community-driven, flexible, and appropriate MDA delivery models will be more effective in reaching and treating mobile populations.
− Integrating MDA with other essential health services (e.g., vaccination campaigns, maternal health) will improve accessibility and coverage.
− The use of digital tools (e.g., mobile tracking, SMS reminders) will enhance MDA follow-up and retention among mobile groups.

#### Operational definitions of concepts.

**Mass drug administration (MDA):** MDA is a campaign strategy in which all eligible people in an area are given preventive chemotherapy regardless of their infection status. MDA is recommended by the WHO and involves periodically treating at-risk populations depending on the level of infection in the community. MDAs are essential for assisting in the control and elimination of NTDs. Achieving WHO-established threshold coverage levels ((1) LF: ≥ 65% of the total population at risk (targeting the entire eligible population); (2) onchocerciasis: ≥ 80% of the eligible population (usually individuals aged 5 years and above in endemic areas); (3) schistosomiasis: ≥ 75% of school-age children (and at-risk adults in some settings); (4) soil-transmitted helminthiases (STH): ≥ 75% of preschool- and school-age children; trachoma: ≥ 80% of the total population in endemic areas (as part of the SAFE strategy, focusing on MDA with azithromycin)) for NTDs elimination is crucial, as success is only guaranteed if the drug reaches the target populations [26,27]

**Mobile populations:** Population mobility is defined as the geographic movement of people from their usual place of residence to another place for different reasons, including looking for better opportunities, to escape disasters and unrest, or safer places [28]. Depending on the rationale for movement, ir can be from one country to another, from one region to another, or between rural and urban areas. Mobile populations include refugees, IDPs, informal economic migrants, nomadic pastoralists and livestock breeders, among others [29,30]. Mobile populations are highly vulnerable because they often lack consistent access to essential services such as healthcare, education, and social protection and hence they face major barriers/obstacles to accessing public health interventions. In the context of this study, we will focus on IDPs, informal economic migrants, nomadic pastoralists including livestock breeders.

**The concept of “effectiveness of MDA”:** In this study, we aim to explore the impact of mobility on MDA implementation. Unlike a systematic review or meta-analysis, we do not intend to conduct our own measurements. Instead, we will summarize existing findings from the literature in a descriptive manner. Based on these findings, we will assess whether a systematic review and meta-analysis are necessary for objective measurement. The primary goal is to map the existing literature on this topic.

## Materials and methods

### Protocol design

We will use the Joanna Briggs Institute methodology (JBI) guidance for scoping reviews to conduct the study [31] and the Preferred Reporting Items for Systematic Review and Meta-Analysis Protocols (PRISMA-P) [32] for writing the protocol (S1 File). The review protocol will not be registered within the International Prospective Register of Systematic Reviews (PROSPERO) because scoping reviews are not currently accepted. We will utilize the PRISMA extension for scoping reviews (PRISMA-ScR) as the reporting guidelines for the scoping review manuscript [33].

### Eligibility criteria

**Methodological framework** for conducting the scoping study: In this paper we will use PCC (Population (or participants)/concept/context) framework to guide the research question and inform the search strategy for our scoping review [34].

**Population:** Our population will include mobile populations, such as those defined above, in regions where MDA for PC-NTDs is being implemented. We will include basic characteristics of participants, such as age, gender, education, economic status.

**Concept/Intervention**: The concept we will be examining in this scoping review is the implementation of MDA for preventive chemotherapy NTDs (PC-NTDs). Health outcomes will include MDA coverage rates, treatment adherence, disease transmission rates, and progress toward NTD elimination. Impact outcomes will include the overall success, easured by coverage rate, of MDA programs, and challenges, such as delays or setbacks in disease control due to missed treatments and reinfection risks.

**Context**: In Sub-Saharan Africa, population mobility severely hampers public health interventions, particularly MDA programs targeting NTDs including lymphatic filariasis, onchocerciasis, trachoma, schistosomiasis, and soil-transmitted helminths [35].

To accomplish the goal of disease elimination, these programs mostly depend on high levels of drug coverage throughout populations [36]. However, in regions where communities are highly mobile due to nomadic lifestyles, seasonal migration, labor migration, or displacement due to conflict or climate change, MDA coverage can be severely impacted [24,37,38]. Geographically, many of these areas in Africa are characterized by remote, hard to reach, rural locations with inadequate health infrastructure and limited access to health care. Efforts to ensure continuity of care along with consistent use of drugs are complicated by the temporary and unpredictable nature of mobile populations, which frequently cross borders internationally [39]. Culturally, some of these populations may have beliefs that can influence their willingness to participate in MDA campaigns. Factors such as language barriers, trust in health systems, and knowledge about NTDs may further exacerbate the challenge. Additionally, this scoping review may also examine how certain sub-groups within these mobile populations such as women, children, or ethnic minorities are differently affected by MDA campaigns [40].

### Inclusion/Exclusion criteria

We will include studies conducted from 2000 until 2024 because, since the 2000s, global efforts to eliminate NTDs such as lymphatic filariasis and onchocerciasis have intensified. International initiatives such as the Global Program for the Elimination of Lymphatic Filariasis (GPELF), launched by WHO in 2000, have marked a turning point in strategies to combat these diseases [41]. The collection of more accurate data on MDA campaigns and population movements has also been enhanced using technological advances, such as Geographic Information Systems (GIS) and health surveillance tools (molecular xenomonitoring) [42,43]. Cross-border migration, humanitarian crises, and refugee and internally displaced person flows have increased, especially after 2000 because of wars in the Middle East and Africa [38].

This study will involve all study designs including randomized controlled trials, non-randomized controlled studies, quasi-experimental, observational studies, and evaluation studies of MDA. Studies published in peer-review journals and found in grey literature will be included. The references cited in the reviewed literature will be searched to identify additional relevant studies. Only studies focusing on Africa will be included. We will include only papers and documents published in English and French.

We will exclude studies that (1) are conducted outside Africa, (2) are prior to the year 2000, (3) are not in French or English, (4) focus on topics unrelated to MDA campaigns, preventive chemotherapy NTD elimination efforts or mobile populations as defined in this protocol.

### Information sources and search strategy

The search strategy will be developed under the guidance of a research librarian of Health Sciences from the University of Ottawa. A comprehensive search strategy using subject headings and keywords will be used to search for potentially eligible published studies in Medline (Ovid), Embase, and Web of Science. An initial search through the Medline (Ovid) database will be conducted using an analysis of text words found in the title and abstract, and the index terms used in describing the article (See a preliminary Medline search: S3 File). Secondly, keywords and index terms shall be identified to search for studies in selected databases. Finally, additional studies not found in the databases shall be searched from the reference lists of the selected studies from the first and second searches.

Additional searches will be conducted on the WHO, World Bank websites and the Centers for Disease Control and Prevention (CDC), The International Organization for Migration (IOM), The International Labour Organization (ILO) and the United Nations High Commissioner for Refugees (UNHCR) to search for unpublished studies. Search terms will include mobile, nomadic populations, migrant, internally displaced person, access to healthcare, neglected tropical diseases, NTDs, Africa, mass drug administration, MDA, care, movement, strategy*.* For grey literature search we will conduct manual searches by reviewing relevant reports, policy briefs, and technical documents available on institutional websites. This will involve navigating organizational repositories, and identifying relevant grey literature that may not be indexed in standard databases.

Zotero software will be used for citation management in this scoping review. The PRESS (Peer Review of Electronic Search Strategies) checklist will be used to ensure the rigor and accuracy of the search strategy for this scoping review.

The search strategy will specifically target the five WHO preventive chemotherapy NTDs of interest using relevant search terms that include but not limited to:

1. **Lymphatic filariasis** or *elephantiasis or filarial or Wuchereria bancrofti or Brugia malayi or Brugia timori or microfilaria* AND
2. **Onchocerciasis** or *onchocerca volvulus* or *river blindness* or *onchocerciasis* or *volvulus* AND
3. **Schistosomiasis** or *Schistosoma haematobium* or *Schistosoma intercalatum or Schistosoma guineensis* or *Schistosoma mansoni* or *bilharzia* or *Katayama fever* or *snail fever* AND
4. **Soil-transmitted helminthiasis (STH)** or *Ascaris lumbricoides* or *Trichuris trichiura* or *Ancylostoma* or *Necator americanus* or *roundworm* or *whipworm* or *hookworm* AND
5. **Trachoma** or *Chlamydia trachomatis* or *trachoma* or *granular conjunctivitis* AND

Moreover, population mobility-related terms such *refugee, pastoralist, cross-border, internally displaced persons (IDPs), economic migrant, transhumance, nomad, migrant population, migrant worker, and population dynamics* will be included to further focus and refine the search.

### Study selection and screening process

Prior to the study’s commencement we will ensure that all team members have a shared interpretation of the inclusion and exclusion criteria, as well as how unclear information will be assessed by multiple team members. In some cases where questions remain concerning a particular study, efforts will be made to contact the authors for clarification to resolve ambiguities.

Studies will be identified in searched databases and saved in Zotero and exported to Covidence for screening. After importing references and inclusion/exclusion criteria into Covidence, two independent reviewers will screen titles and abstracts of included studies. All conflicts between the two reviewers will be resolved by a third reviewer. Once potentially eligible studies have been selected, this same procedure will be applied for full text screening. We will hold a briefing session with all reviewers on the screening process and the steering of inclusion/exclusion criteria and conflict resolution. We will be meeting regularly to discuss progress, address any difficulties, and explore ways to collaborate effectively.

### Assessment of study quality

Unlike systematic reviews, a formal quality assessment is optional in scoping reviews, which are intended to map the extent and nature of evidence on a broad topic. However, the team will assess and discuss the quality of the studies informally, in order to avoid giving undue weight to the results of what we consider to be a lower quality study. For example, if a study is purely descriptive of only a few people or does not describe aspects of the study in a way considered rigorous by the team, we will highlight this when reporting on that study

### Data charting, collection and extraction procedures

Two independent reviewers will extract data using a Microsoft Excel spreadsheet adopted from the Joanna Briggs Institute’s template for data extraction (S2 File) [44,45]. The template will be piloted on three articles before use. The following information will be gathered: (i) study characteristics, such as the name of the first authors, the year of publication, the country, and the study design; (ii) details about interventions that were put into place, such as implementation strategies, factors that helped or hindered the implementation of rural pipeline programs, and the results or impacts of those programs; (iii) reasons for movement and patterns of movement of mobile populations, the consequences on health outcomes and the approaches being developed to overcome these issues among mobile and migrant populations. In line with our review questions, both quantitative and qualitative data will be collected and analyzed. We will resolve discrepancies by consensus, or a third reviewer will be required. The rules for data extraction will be documented and stored with the protocol and template at the research data storage facility of ICERMali at the University of Sciences, Techniques and Technologies of Bamako (USTTB) in Mali.

We will include specific data extraction fields focusing on mobile populations, MDA interventions, and effectiveness outcomes. For mobile populations, fields will capture demographic data (e.g., age, gender, socio-economic status), migration patterns (e.g., seasonal, nomadic, cross-border movement), and risk factors (e.g., exposure to disease vectors, access to healthcare). For MDA interventions, we will document intervention types (e.g., mass drug administration, targeted treatment), delivery methods (e.g., door-to-door, fixed-site, mobile teams), coverage rates (e.g., proportion of population reached), and community engagement strategies (e.g., awareness campaigns, local health worker involvement). Effectiveness outcomes will include treatment compliance rates, disease prevalence reduction, and any reported adverse effects or treatment failures.

### Synthesis and presentation of results

Extracted data will be reported in tables and/or figures. Further formats can be considered at the time of analysis. The results of this scoping review will be descriptive, including an explanation of their study characteristics with reference to study population on which they focus.

We will summarize the characteristics of included studies in a descriptive way. With an emphasis on mapping important concepts, populations, and features, we will compile and display the extracted data descriptively according to JBI recommendations [46]. We will report the results in text, tables and graphs following PRISMA-ScR guidelines.

### Expected outcomes of the study

It is expected that the results of this study will reveal a low proportion of non-compliance and access to MDA among mobile and migrant populations. It is also expected that the results will reveal a relationship between the socio-demographic/structural characteristics of participants and MDA participation among mobile populations in Africa. We also anticipate that this study will determine why some people missed treatment and what recommendations are needed to improve MDA coverage rates in MMPs in Africa We will attempt to highlight evidence from the literature on the health status of mobile populations and to assess the best ways to provide them with MDA campaigns.

### Potential limitations

This review may have limitations related to the search strategy. The inclusion of only studies published in French and/or English, as well as the specific search terms used, may reduce the sensitivity in identifying relevant studies. This study may also suffer from publication bias.

We will work with stakeholders and experts, if necessary, to find unpublished studies and incorporate studies in English and French to capture the most relevant content in order to reduce publication and language bias. The inclusion of studies in English and French is justified by the fact that these languages are frequently used in worldwide research, especially in the study’s target regions, like West Africa, guaranteeing thorough coverage of important studies. Furthermore, the writers’ fluency in both languages enables proper literature interpretation and analysis.

### Ethics statement and dissemination of results

This protocol is part of a thesis protocol. We received ethical clearance from the University of Sciences, Techniques, and Technologies of Bamako’s Ethical Review Committee (Approval No. 2023/11/CE/USTTB), Mali, and from the Health Sciences and Sciences Research Ethics Board of the University of Ottawa (Approval No. H- 02- 23- 8759). This specific scoping review relies on studies already published or in the public domain and therefore does not require ethical approval. However, all measures will be taken to respect the procedures of scientific integrity.

The results of this study will be used in collaboration with program managers to strengthen the elimination or control of NTDs in Africa. The dissemination of the results of the scoping review will be done through peer review publication, presentation at conferences and other relevant stakeholder fora.

### Status and timeline of the study

The scoping review will be conducted over a six-month period, from February to July 2025 (Table 1).

**Table 1:** Activities and timeline of the study.

Activities	February 2025	March 2025	April 2025	May 2025	June 2025	July 2025
Search strategy developpement						
Screening of studies and analysis of titles and abstracts						
Selection of studies and analysis of studies in full texts						
Data extraction						
Data analysis and discussion						
Presentation of results						
Scope review submission						

## Conclusion

Our review attempts to address the social inequalities faced by populations in Africa. Mass treatment is an effective way to prevent disease, but to be effective it is necessary that they be implemented and adapted to different contexts and population groups, especially those most hard to reach. The aim of this review is to explore the influences of population mobility on the implementation of MDA in Africa, and to examine the evidence of factors influencing movement and approaches that have proven effective in improving MDA coverage among them. Our review will answer these questions across Africa, thus helping to improve mass treatments and increase health equity for all. Further, other disease control and elimination programs in Africa and elsewhere that are using mass treatment methods will potentially benefit from our results. Similarly, the information generated will contribute to reaching the global elimination targets.

## Supporting information

S1 File Completed PRISMA-P 2015 checklist. (DOCX)

S2 File Data extraction sheet information. (DOCX)

S3 File Preliminary search from Medline. (DOCX)

## Abbreviations:

ABS: Australian Bureau of Statistics
CDC: Centers for Disease Control and Prevention
GIS: Geographic Information Systems
GPELF: Global Program for the Elimination of Lymphatic Filariasis
IDPs: Internally Displaced Persons
JBI: Joanna Briggs Institute methodology
LF: Lymphatic Filariasis
MDA: Mass Drug Administration
MMPs: Mobile and Migrant Populations
NTDs: Neglected Tropical Diseases
PRESS: Peer Review of Electronic Search Strategies
PRISMA-P: Preferred Reporting Items for Systematic Reviews and Meta-Analysis Protocols
PC-NTDs: Preventive Chemotherapy NTDs
PRISMA-ScR: PRISMA Extension for Scoping Reviews
STPH: Swiss Tropical And Public Health Institute
ILO: The International Labour Organization
IOM: The International Organization for Migration
PROSPERO: The International Prospective Register of Systematic reviews
USAID: The United States Agency for International Development
UNHCR: United Nations High Commissioner for Refugees
USTTB: Université des Sciences, des Techniques et des Technologies de Bamako
WHO: World Health Organization

## References

[1] WHO. Neglected tropical diseases -- GLOBAL [Internet]; 2024 [cited 2024 Dec 22]. Available from: https://www.who.int/health-topics/neglected-tropical-diseases

[2] Álvarez-Hernández D-A, Rivero-Zambrano L, Martínez-Juárez L-A, García-Rodríguez-Arana R. Overcoming the global burden of neglected tropical diseases. Ther Adv Infect Dis. 2020;7:2049936120966449. doi: 10.1177/2049936120966449 33178435 PMC7592315

[3] Medeiros Z, Vieira A, Xavier A, Bezerra G, Lopes M, Bonfim C. Lymphatic filariasis: a systematic review on morbidity and its repercussions in countries in the Americas. Int J Environ Res Public Health. 2021;19(1). 10.3390/ijerph19010316 PMC8751179 35010576

[4] Gómez LJ, van Wijk R, van Selm L, Rivera A, Barbosa MC, Parisi S, et al. Stigma, participation restriction and mental distress in patients affected by leprosy, cutaneous leishmaniasis and Chagas disease: a pilot study in two co-endemic regions of eastern Colombia. Trans R Soc Trop Med Hyg. 2020;114(7):476–82. doi: 10.1093/trstmh/trz132 32052043 PMC7334822

[5] Orish VN, Morhe EKS, Azanu W, Alhassan RK, Gyapong M. The parasitology of female genital schistosomiasis. Curr Res Parasitol Vector Borne Dis. 2022;2:100093. doi: 10.1016/j.crpvbd.2022.100093 35719849 PMC9198370

[6] Vlassoff C, Arogundade K, Patel K, Jacobson J, Gyapong M, Krentel A. Improving the response of health systems to female genital schistosomiasis in endemic countries through a gender-sensitive human rights-based framework. Diseases. 2022;10(4):125. doi: 10.3390/diseases10040125 36547211 PMC9777435

[7] Lenk EJ, Redekop WK, Luyendijk M, Rijnsburger AJ, Severens JL. Productivity loss related to neglected tropical diseases eligible for preventive chemotherapy: a systematic literature review. PLoS Negl Trop Dis. 2016;10(2):e0004397. doi: 10.1371/journal.pntd.0004397 26890487 PMC4758606

[8] Peeling RW, Boeras DI, Nkengasong J. Re-imagining the future of diagnosis of Neglected Tropical Diseases. Comput Struct Biotechnol J. 2017;15:271–4. doi: 10.1016/j.csbj.2017.02.003 28352456 PMC5358522

[9] King IL, Li Y. Host–parasite interactions promote disease tolerance to intestinal helminth infection. Front Immunol [Internet]. 2018;9:2128. Available from: https://www.frontiersin.org/article/10.3389/fimmu.2018.02128/full 30298071 10.3389/fimmu.2018.02128 PMC6160735

[10] Krentel A, Basker N, Beau de Rochars M, Bogus J, Dilliott D, Direny AN, et al. A multicenter, community-based, mixed methods assessment of the acceptability of a triple drug regimen for elimination of lymphatic filariasis. PLoS Negl Trop Dis. 2021;15(3):e0009002. doi: 10.1371/journal.pntd.0009002 33657090 PMC7928496

[11] WHO. Global update on implementation of preventive chemotherapy (PC) against neglected tropical diseases (NTDs) in 2022 and status of donated medicines for NTDs in 2022–2023 [Internet]; 2023 [cited 2024 Nov 2]. Available from: https://www.who.int/publications/i/item/who-wer9852-681-696

[12] WHO. Global programme to eliminate lymphatic filariasis: progress report, 2022. Wkly Epidemiol Rec;41.

[13] Chiphwanya J, Mkwanda S, Kabuluzi S, Mzilahowa T, Ngwira B, Matipula DE, et al. Elimination of lymphatic filariasis as a public health problem in Malawi. PLoS Negl Trop Dis. 2024;18(2):e0011957. doi: 10.1371/journal.pntd.0011957 38363794 PMC10903958

[14] WHO. Validation of elimination of lymphatic filariasis as a public health problem [Internet]. World Health Organization; 2017 [cited 2024 Dec 27]. Available from: https://iris.who.int/handle/10665/254377

[15] WHO. Weekly Epidemiological Record, 2024, vol. 99, 40 [full issue]. Vol. 99, Weekly Epidemiological Record = Relevé épidémiologique hebdomadaire. World Health Organization = Organisation mondiale de la Santé; 2024. p. 565–76.

[16] WHO. Benin and Mali eliminate trachoma as a public health problem | ESPEN [Internet]; 2023 [cited 2024 Oct 17]. Available from: https://espen.afro.who.int/updates-events/updates/benin-and-mali-eliminate-trachoma-as-a-public-health-problem

[17] Dolo H, Coulibaly ME, Sow M, Coulibaly YI, Doumbia M, Sangare M, et al. Progress towards elimination of onchocerciasis transmission in Mali: a “pre-stop MDA” survey in 18 transmission zones. PLoS Negl Trop Dis. 2023;17(11):e0011632. doi: 10.1371/journal.pntd.0011632 37967137 PMC10686495

[18] Jacobson J, Pantelias A, Williamson M, Kjetland EF, Krentel A, Gyapong M, et al. Addressing a silent and neglected scourge in sexual and reproductive health in Sub-Saharan Africa by development of training competencies to improve prevention, diagnosis, and treatment of female genital schistosomiasis (FGS) for health workers. Reprod Health. 2022;19(1):20. doi: 10.1186/s12978-021-01252-2 35073965 PMC8785555

[19] USAID. Mali develops sustainability plan as they advance towards elimination and control of targeted neglected tropical diseases. 2022.

[20] Dean L, Ozano K, Adekeye O, Dixon R, Fung EG, Gyapong M, et al. Neglected tropical diseases as a “litmus test” for universal health coverage? Understanding who is left behind and why in Mass Drug Administration: lessons from four country contexts. PLoS Negl Trop Dis. 2019;13(11):e0007847. doi: 10.1371/journal.pntd.0007847 31751336 PMC6871774

[21] IOM. Who is a Migrant? | IOM Blog [Internet]. [cited 2024 Dec 25]. Available from: https://weblog.iom.int/who-migrant

[22] Camlin CS, Akullian A, Neilands TB, Getahun M, Eyul P, Maeri I, et al. Population mobility associated with higher risk sexual behaviour in eastern African communities participating in a Universal Testing and Treatment trial. J Int AIDS Soc. 2018;21 Suppl 4(Suppl Suppl 4):e25115. doi: 10.1002/jia2.25115 30027668 PMC6053476

[23] Camlin CS, Cassels S, Seeley J. Bringing population mobility into focus to achieve HIV prevention goals. J Int AIDS Soc. 2018;21 Suppl 4(Suppl Suppl 4):e25136. doi: 10.1002/jia2.25136 30027588 PMC6053544

[24] Sangare M, Diabate AF, Coulibaly YI, Tanapo D, Thera SO, Dolo H, et al. Understanding the barriers and facilitators related to never treatment during mass drug administration among mobile and migrant populations in Mali: a qualitative exploratory study. BMJ Glob Health. 2024;9(10):e015671. doi: 10.1136/bmjgh-2024-015671 39384331 PMC11474861

[25] McMichael C. Climate change-related migration and infectious disease. Virulence. 2015;6(6):548–53. doi: 10.1080/21505594.2015.1021539 26151221 PMC4720222

[26] WHO. Mass Drug Administration (MDAs): an opportunity for community engagement and social behaviour change [Internet]; 2024 [cited 2024 Oct 18]. Available from: https://www.who.int/publications/m/item/mass-drug-administration-(mdas)--an-opportunity-for-community-engagement-and-social-behaviour-change

[27] WHO. Crossing the Billion. Preventive chemotherapy for neglected tropical diseases [Internet]; 2017 [cited 2025 Apr 7]. Available from: https://www.who.int/publications/i/item/9789240696471

[28] ABS. Population mobility. Canberra: Australian Bureau of Statistics; 2009.

[29] Scheel S, Tazzioli M. Who is a migrant? Abandoning the nation-state point of view in the study of migration. Mig Pol [Internet]. 2022;1(1):002. Available from: doi: 10.21468/migpol.1.1.002

[30] STPH. Mobile populations [Internet]. Swiss TPH; 2024 [cited 2024 Dec 24]. Available from: https://www.swisstph.ch/en/topics/society-and-health/mobile-populations

[31] Peters MDJ, Marnie C, Tricco AC, Pollock D, Munn Z, Alexander L, et al. Updated methodological guidance for the conduct of scoping reviews. JBI Evid Synth. 2020;18(10):2119–26. doi: 10.11124/JBIES-20-00167 33038124

[32] Shamseer L, Moher D, Clarke M, Ghersi D, Liberati A, Petticrew M, et al. Preferred reporting items for systematic review and meta-analysis protocols (PRISMA-P) 2015: elaboration and explanation. BMJ. 2015;350:g7647. doi: 10.1136/bmj.g7647 25555855

[33] Tricco AC, Lillie E, Zarin W, O’Brien KK, Colquhoun H, Levac D, et al. PRISMA extension for scoping reviews (PRISMA-ScR): checklist and explanation. Ann Intern Med. 2018;169(7):467–73. doi: 10.7326/M18-0850 30178033

[34] Pollock D, Peters MDJ, Khalil H, McInerney P, Alexander L, Tricco AC, et al. Recommendations for the extraction, analysis, and presentation of results in scoping reviews. JBI Evid Synth. 2023;21(3):520–32. doi: 10.11124/JBIES-22-00123 36081365

[35] Merrill R, Chabi A, McIntyre E, Kouassi J, Alleby M, Codja C. An approach to integrate population mobility patterns and sociocultural factors in communicable disease preparedness and response. Humanit Soc Sci Commun [Internet]. 2021;8(1):23. Available from: https://www.nature.com/articles/s41599-020-00704-7 PMC11010577 38617731

[36] Silumbwe A, Halwindi H, Zulu JM. How community engagement strategies shape participation in mass drug administration programmes for lymphatic filariasis: the case of Luangwa District, Zambia. PLoS Negl Trop Dis. 2019;13(11):e0007861. doi: 10.1371/journal.pntd.0007861 31774820 PMC6905562

[37] Muchiri G, Okwii M, Bukuluki P, Willems J, Amanyi-Enegela J, Yibi M. Challenges and strategies for the uptake of mass drug administration among pastoralist communities in South Sudan. Front Trop Dis [Internet]. 2023;4:1007480. Available from: https://www.frontiersin.org/articles/10.3389/fitd.2023.1007480/full

[38] Zimmerman C, Kiss L, Hossain M. Migration and health: a framework for 21st century policy-making. PLoS Med. 2011;8(5):e1001034. doi: 10.1371/journal.pmed.1001034 21629681 PMC3101201

[39] Adams MW, Sutherland EG, Eckert EL, Saalim K, Reithinger R. Leaving no one behind: targeting mobile and migrant populations with health interventions for disease elimination-a descriptive systematic review. BMC Med. 2022;20(1):172. doi: 10.1186/s12916-022-02365-6 35527246 PMC9082871

[40] George NS, David SC, Nabiryo M, Sunday BA, Olanrewaju OF, Yangaza Y, et al. Addressing neglected tropical diseases in Africa: a health equity perspective. Glob Health Res Policy. 2023;8(1):30. doi: 10.1186/s41256-023-00314-1 37491338 PMC10367333

[41] Turner HC, Ottesen EA, Bradley MH. A refined and updated health impact assessment of the Global Programme to Eliminate Lymphatic Filariasis (2000-2020). Parasit Vectors. 2022;15(1):181. doi: 10.1186/s13071-022-05268-w 35643508 PMC9148484

[42] Fletcher-Lartey SM, Caprarelli G. Application of GIS technology in public health: successes and challenges. Parasitology. 2016;143(4):401–15. doi: 10.1017/S0031182015001869 26831619

[43] Riches N, Badia-Rius X, Mzilahowa T, Kelly-Hope LA. A systematic review of alternative surveillance approaches for lymphatic filariasis in low prevalence settings: implications for post-validation settings. PLoS Negl Trop Dis. 2020;14(5):e0008289. doi: 10.1371/journal.pntd.0008289 32396575 PMC7217451

[44] JBI. 10.3.7.3 Data extraction - JBI manual for evidence synthesis - JBI Global Wiki [Internet]; 2024 [cited 2024 Dec 27]. Available from: https://jbi-global-wiki.refined.site/space/MANUAL/355863071/10.3.7.3+Data+extraction

[45] Peters MD, Godfrey C, McInerney P, Munn Z, Tricco AC, Khalil H. Scoping reviews. In: Aromataris E, Lockwood C, Porritt K, Pilla B, Jordan Z, editors. JBI manual for evidence synthesis [Internet]. JBI; 2024 [cited 2024 Dec 27]. Available from: https://jbi-global-wiki.refined.site/space/MANUAL/355862497/10.+Scoping+reviews

[46] Aromataris E, Lockwood C, Porritt K, Pilla B, Jordan Z, editors. JBI manual for evidence synthesis [Internet]. JBI; 2024 [cited 2024 Oct 17]. Available from: https://jbi-global-wiki.refined.site/space/MANUAL

