# Supplementary material for: Exploring the impact of mobile and migrant populations on mass drug administration coverage and effectiveness in Africa: A scoping review protocol
Source: PLoS One. 2025 May 29;20(5):e0324949. doi: 10.1371/journal.pone.0324949 (PMC12121756; doi:10.1371/journal.pone.0324949)
Supplement: S1 File — (DOCX) [file pone.0324949.s001.docx]

**Supplemental 1.**

PRISMA-P (preferred reporting items for systematic review and meta-analysis protocols) 2015 checklist: recommended items to address in a systematic review protocol

| **Section and topic** | **Item No** | **Checklist item** | **REPORTED ON PAGE #** |
| --- | --- | --- | --- |
| **Administrative information** | | |  |
| Title: |  |  |  |
| Identification | 1a | Identify the report as a protocol of a systematic review | The study is identified in the title as a scoping review protocol |
| Update | 1b | If the protocol is for an update of a previous systematic review, identify as such | This is not an update to a previous review |
| Registration | 2 | If registered, provide the name of the registry (such as PROSPERO) and registration number | No. The protocol was not registered |
| Authors: |  |  |  |
| Contact | 3a | Provide name, institutional affiliation, e-mail address of all protocol authors; provide physical mailing address of corresponding author | This information is on page 1 and 15. |
| Contributions | 3b | Describe contributions of protocol authors and identify the guarantor of the review | Author contributions and funding are included in the manuscript on page 15 and 16. |
| Amendments | 4 | If the protocol represents an amendment of a previously completed or published protocol, identify as such and list changes; otherwise, state plan for documenting important protocol amendments | The protocol does not represent an amendment to a previously completed or published protocol. |
| Support: |  |  |  |
| Sources | 5a | Indicate sources of financial or other support for the review | This review is part of a PhD thesis protocol and received no funding. |
| Sponsor | 5b | Provide name for the review funder and/or sponsor | This review is part of a PhD thesis protocol and received no funding. |
| Role of sponsor or funder | 5c | Describe roles of funder(s), sponsor(s), and/or institution(s), if any, in developing the protocol | Not applicable |
| **Introduction** | | |  |
| Rationale | 6 | Describe the rationale for the review in the context of what is already known | Yes. The review rationale is on pages 3 to 6 of the manuscript. |
| Objectives | 7 | Provide an explicit statement of the question(s) the review will address with reference to participants, interventions, comparators, and outcomes (PICO) | The review questions/objectives are on pages 5 and 6. Yes. The key elements (population or participants, concepts, and context) are on pages 7 and 8 of the manuscript. |
| **Methods** | | |  |
| Eligibility criteria | 8 | Specify the study characteristics (such as PICO, study design, setting, time frame) and report characteristics (such as years considered, language, publication status) to be used as criteria for eligibility for the review | Yes. The eligibility criteria is on pages 7, 8 and 9 of the manuscript. |
| Information sources | 9 | Describe all intended information sources (such as electronic databases, contact with study authors, trial registers or other grey literature sources) with planned dates of coverage | Yes, this information is included on page 10 of the manuscript. |
| Search strategy | 10 | Present draft of search strategy to be used for at least one electronic database, including planned limits, such that it could be repeated | Yes, this information is included on page 10 of the manuscript. |
| Study records: |  |  |  |
| Data management | 11a | Describe the mechanism(s) that will be used to manage records and data throughout the review | Yes. The mechanism(s) that will be used to manage records and data during the review are presented on page 11 and 12 of the manuscript. |
| Selection process | 11b | State the process that will be used for selecting studies (such as two independent reviewers) through each phase of the review (that is, screening, eligibility and inclusion in meta-analysis) | The process for selecting sources of evidence is on page 11. |
| Data collection process | 11c | Describe planned method of extracting data from reports (such as piloting forms, done independently, in duplicate), any processes for obtaining and confirming data from investigators | Yes, the methods of collection process data are on pages 11 and 12. |
| Data items | 12 | List and define all variables for which data will be sought (such as PICO items, funding sources), any pre-planned data assumptions and simplifications | Yes. The list and definition are on supplemetal table 2. |
| Outcomes and prioritization | 13 | List and define all outcomes for which data will be sought, including prioritization of main and additional outcomes, with rationale | Yes, this information is included on page 12 of the manuscript. |
| Risk of bias in individual studies | 14 | Describe anticipated methods for assessing risk of bias of individual studies, including whether this will be done at the outcome or study level, or both; state how this information will be used in data synthesis | Not applicable to this study |
| Data synthesis | 15a | Describe criteria under which study data will be quantitatively synthesised | Not applicable to this study |
|  | 15b | If data are appropriate for quantitative synthesis, describe planned summary measures, methods of handling data and methods of combining data from studies, including any planned exploration of consistency (such as I^2^, Kendall’s τ) | Not applicable to this study |
|  | 15c | Describe any proposed additional analyses (such as sensitivity or subgroup analyses, meta-regression) | Not applicable to this study |
|  | 15d | If quantitative synthesis is not appropriate, describe the type of summary planned | Yes, this information can be found on page 12 of the manuscript. |
| Meta-bias(es) | 16 | Specify any planned assessment of meta-bias(es) (such as publication bias across studies, selective reporting within studies) | Not applicable |
| Confidence in cumulative evidence | 17 | Describe how the strength of the body of evidence will be assessed (such as GRADE) | Not applicable |

**From:** Shamseer L, Moher D, Clarke M, Ghersi D, Liberati A, Petticrew M, Shekelle P, Stewart L, PRISMA-P Group. Preferred reporting items for systematic review and meta-analysis protocols (PRISMA-P) 2015: elaboration and explanation. BMJ. 2015 Jan 2;349(jan02 1):g7647. (<https://www.bmj.com/content/349/bmj.g7647.long>)
