# Supplementary material for: Exploring the impact of mobile and migrant populations on mass drug administration coverage and effectiveness in Africa: A scoping review protocol
Source: PLoS One. 2025 May 29;20(5):e0324949. doi: 10.1371/journal.pone.0324949 (PMC12121756; doi:10.1371/journal.pone.0324949)
Supplement: S2 File — (DOCX) [file pone.0324949.s002.docx]

**Supplemental 2**Appendix 10.1 JBI template source of evidence details, characteristics and results extraction instrument

| **Scoping review details** | |
| --- | --- |
| Scoping Review title: |  |
| Review objective/s: |  |
| Review question/s: |  |
| **Inclusion/Exclusion Criteria** | |
| Population |  |
| Concept |  |
| Context |  |
| Types of evidence source |  |
| **Evidence source Details and Characteristics** | |
| Citation details (e.g. author/s, date, title, journal, volume, issue, pages) |  |
| Country |  |
| Context |  |
| Participants (details e.g. age/sex and number) |  |
| **Details/Results extracted from source of evidence**(in relation to the concept of the scoping review) | |
| E.g. Quality of Life Domains assessed |  |
| E.g. Number of items in tool |  |
| E.g. details of psychometric validation of tool |  |
