# Supplementary material for: Exploring the impact of mobile and migrant populations on mass drug administration coverage and effectiveness in Africa: A scoping review protocol
Source: PLoS One. 2025 May 29;20(5):e0324949. doi: 10.1371/journal.pone.0324949 (PMC12121756; doi:10.1371/journal.pone.0324949)
Supplement: S3 File — (DOCX) [file pone.0324949.s003.docx]

**Preliminary search strings (search query from Medline)**
Ovid MEDLINE(R) ALL <1946 to February 11, 2025>

| **#** | **Query** | **Results from 11 Feb 2025** |
| --- | --- | --- |
| 1 | exp Population Dynamics/ | 86,297 |
| 2 | population dynamic*.ti,ab,kf. | 42,163 |
| 3 | exp "Emigration and Immigration"/ or exp "Emigrants and Immigrants"/ or exp "Transients and Migrants"/ | 53,448 |
| 4 | (Migrant? or Transient? or Emigrant? or Immigrant? or refugee?).ti,ab,kf. | 418,779 |
| 5 | exp Human Migration/ | 28,343 |
| 6 | Refugees/ or Refugee Camps/ | 14,616 |
| 7 | Cross-border population*.ti,ab,kf. | 29 |
| 8 | (Internally displaced adj2 (person* or people or population)).ti,ab,kf. | 865 |
| 9 | (pastoral* or nomad* or transhum*).ti,ab,kf. | 7,906 |
| 10 | Travel pattern*.ti,ab,kf. | 469 |
| 11 | ("no one behind" or ("no one" adj2 "left behind")).ti,ab,kf. | 403 |
| 12 | or/1-11 | 527,668 |
| 13 | exp Filariasis/ or exp Elephantiasis, Filarial/ or exp Mass Drug Administration/ or exp Albendazole/ | 21,592 |
| 14 | ((mass adj2 (administration* or treatment)) or NTD program*).ti,ab,kf. | 5,750 |
| 15 | exp Trachoma/ or exp Ivermectin/ or exp Onchocerciasis/ or exp Schistosomiasis/ or exp Anthelmintics/ | 99,886 |
| 16 | (Mass Chemotherap* or Preventive chemotherap*).ti,ab,kf. | 1,048 |
| 17 | (Health adj2 (intervention* or system?)).ti,ab,kf. | 201,133 |
| 18 | exp Neglected Diseases/ or exp Helminthiasis/ or exp Schistosomiasis/ or exp Tropical Medicine/ | 145,464 |
| 19 | (Disease? adj2 (elimination or control or eradication)).ti,ab,kf. | 87,884 |
| 20 | Neglected Tropical disease?.ti,ab,kf. | 6,471 |
| 21 | Lymphatic filariasis*.ti,ab,kf. | 3,439 |
| 22 | Onchocerciasis*.ti,ab,kf. | 4,305 |
| 23 | Schistosomiasis*.ti,ab,kf. | 20,924 |
| 24 | Soil-transmitted helminth*.ti,ab,kf. | 2,543 |
| 25 | Trachoma*.ti,ab,kf. | 20,103 |
| 26 | Parasitic infection*.ti,ab,kf. | 11,220 |
| 27 | Vector-borne disease*.ti,ab,kf. | 6,090 |
| 28 | or/13-27 | 520,095 |
| 29 | Africa/ or exp Africa South of the Sahara/ | 308,605 |
| 30 | (africa or Angola or Congo or Ghana or Mauritius or Sierra Leone or Benin or Cote d'Ivoire or Guinea or Somalia or Botswana or Guinea-Bissau or Mozambique or South Africa or Burkina Faso or Djibouti or Kenya or Namibia or Sudan or Burundi or Equatorial Guinea or Lesotho or Niger or Cabo Verde or Eritrea or Liberia or Nigeria or Togo or Cameroon or Eswatini or Madagascar or Rwanda or Tanzania or Central African or Ethiopia or Malawi or Sao Tome or Uganda or Chad or Gabon or Mali or Senegal or Zambia or Comoros or Gambia or Mauritania or Seychelles or Zimbabwe).ti,ab,kf. | 500,194 |
| 31 | 29 or 30 | 574,265 |
| 32 | 12 and 28 and 31 | 2,104 |
| 33 | limit 32 to (english or french) | 1,994 |
| 34 | limit 33 to yr="2000 -Current" | 1,508 |
| 35 | (Understanding the barriers and facilitators related to never treatment during mass drug administration among mobile and migrant populations in Mali).m_titl. | 1 |
| 36 | (Leaving no one behind: targeting mobile and migrant populations with health interventions).m_titl. | 1 |
| 37 | "Addressing neglected tropical diseases in Africa: a health equity perspective.".m_titl. | 1 |
| 38 | 35 or 36 or 37 | 3 |
| 39 | 34 and 38 | 3 |
